# Should the Marriage Contract Be Limited by Law?1Read at the Buffalo Academy of Medicine, February 9, 1897.

**Published:** 1897-05

**Authors:** E. T. Rulison

**Affiliations:** Buffalo, N. Y. 37 Elmwood Avenue


### SHOULD THE MARRIAGE CONTRACT BE LIMITED BY LAW ?[1]

By E. T. RULISON, M. D., Buffalo, N. Y.

"THERE is a destiny made for a man by his ancestors, and no one can elude the tyranny of his organisation." These were the words of Maudsley, and truer, more impressive ones were never uttered. In taking the affirmative side of this question, Should the marriage contract be limited by law ? I do so realising fully that public sentiment, formed and fostered by writers of poetry and fiction, would be shocked to have such a measure proposed for legal enactment. Still I know that in the every-day experience of the physician are to be found many reasons why it should be. The history of marriage is very obscure. It is supposed to date back to the time when a virtuous woman in the tribe to which she belonged was not esteemed or looked upon with favor, but when captured by a man of a neighboring tribe was expected and required to be his property only. In time, this requirement or contract, called marriage, was adopted by the men and women of the same tribe. The oracles, or priests, assumed or usurped the office of performing the marriage ceremony, which office they have continued to hold, with unsurpassed regularity, down to the present time, notwithstanding the many changes in administration and forms of government.

There is really no law regarding the contract of marriage—the consent of the contracting parties alone being sufficient to make it lawful. The marriages prohibited by the Jewish canonical law may be divided into the following : (1) Prohibitions on account of consanguinity and affinity ; (2) prohibitions in consideration of chastity ; (3) prohibitions for religious and other reasons. These reasons are uncertainty of parentage, pregnancy, mourning, consent of parties, mental capacity and legal age. Idiocy and lunacy, coming under the head of mental capacity, are prohibited ; so are spadones, if violently or artificially emasculated, but if this defect be a natural one it does not preclude the contract of a legal marriage. In regard to defects in consequence of disease, or the disease itself, there is a difference of opinion among the rabinical authorities, but what they are I have been unable to learn. The intermarriage of Jews with Gentiles is not in accordance with rabinical law, but when the ceremony is performed by civil authorities the church

1. Read at the Buffalo Academy of Medicine, February 9, 1897.

recognises the contract as legal. This law is rarely infracted, which accounts for the marked differences that exist between the two races.

The reasons for having some law regulating marriages are many, no matter how difficult it might be to have such a law enacted, and especially enforced. Parentage is too serious a question to leave to the hap-hazard way now prevalent regarding it. It is not necessary to discuss at length the question of heredity. Dr. Strahan says, "It is that mysterious influence which foreordains that the offspring shall be in the likeness of its parents." If there be any present who have any doubts regarding the transmission of pathological as well as physiological and psychological traits, I would respectfully refer them to Ribot's work on Heredity. When one comes to study statistics concerning the increasing prevalence of crime, insanity and idiocy, and finds that it is due largely to our careless way of allowing like to beget like, he may well feel alarmed, for he cannot help seeing what the ultimate result will be unless some radical measure be adopted to prevent. Dr. Maudsley observes:

> Because it has been the fashion to look upon an individual as if he were the product of an independent creative act and a self-sufficient being—because men commonly look not beyond a single link in the chain of causation—therefore, it has been impossible hitherto to uproot the erroneous notion, explicitly declared or implicitly held, that each one is endowed by nature with a certain fixed mental potentiality of uniform character. But now that observation reveals more and more clearly every day how much the capacity and character, mental and bodily, of the individual is dependent upon his ancestral antecedents, it is impossible to deny that a man may suffer irremediable ill through the misfortune of bad descent. Each one is a link in the chain of organic beings, a physical consequent of physical antecedents; the idiot is not an accident, nor the irreclaimable criminal an unaccountable casualty.

It seems strange, when this great fundamental law of hereditary transmission has been understood for years, and has been applied to animals, (as well as to the human race, when dollars and cents were concerned,) yet it is ignored entirely when we see children begotten every year with pedigrees which would send our domestic animals to the abattoir or place them under the ban of a health commissioner. Darwin says:

> Man scans with scrupulous care the character and pedigree of his horses, cattle and dogs before he matches them, but when he comes to his own marriage he rarely or never takes any such care. He is impelled by nearly the same motives as the lower animals when they are left to their own free choice, though he is so far superior to them that he highly values mental charms and virtues. On the other hand, he is strongly attracted by mere wealth or rank. Yet he might by selection do something, not only for the bodily constitution and frame of his offspring, but for their intellectual and moral qualities. Both sexes ought to refrain from marriage if they are in any marked degree inferior in body or mind; but such hopes are Utopian, and will never be even partially realised until the laws of inheritance are thoroughly understood. Every one does good service who aids toward this end. When the principles of breeding and inheritance are better understood, we shall not hear ignorant members of our legislature rejecting with scorn a plan for ascertaining whether or not consanguineous marriages are injurious to man.

It was a well-known principle among the Greeks to select their wives with a view to the health and vigor of their children. The Grecian poet, Theogius, who lived 550 B. C., clearly saw the importance of proper sexual selection when he wrote as follows:

With kine and horses, Kernus, we proceed
By reasonable rules, and choose a breed
For profit and increase, at any price:
Of a sound stock, without defect or vice,
But in the daily matches that we make,
The price is everything: for money's sake,
Men marry women; women are in marriage given;
The churl or ruffian, that in wealth has thriven,
May match his offspring with the proudest race;
Thus everything is mixed, noble and base!
If then in outward manner, form and mind,
You find us a degraded, motley kind,
Wonder no more, my friend, the cause is plain,
And to lament the consequence is vain.

One of the greatest obstacles to our reaching a more ideal civilisation is the fact that the low, diseased and vicious marry at an early age and beget many offspring, whilst the careful and virtuous marry later in life and have comparatively few children. Mr. Greg says:

The careless, squalid, unaspiring Irishman multiplies like rabbits; the frugal, foreseeing, self-respecting, ambitious Scot, stern in his morality, spiritual in his faith, sagacious and disciplined in his intelligence, passes his best years in struggle and in celibacy, marries late and leaves few behind him. Given a land originally peopled by a thousand Saxons and a thousand Celts and in a dozen generations five-sixths of the population would be Celts, but five-sixths of the property, of the power of the intellect would belong to the one-sixth of the Saxons that remained. In the eternal struggle for existence it would be the inferior and less favored race that had prevailed—and prevailed by virtue, not of its good qualities, but of its faults.

It has been the history of all newly-discovered countries that progress has been most rapid on account of the "daring and persistent energy" of the settlers. They are generally composed of the most healthy and robust members of the country from which they emigrated, and until the succeeding generations are weakened by vice, intemperance and disease, there follow the natural results of proper selection. With savages, the weak in mind and body are soon eliminated, and those who survive exhibit increased, vigorous health. We, as a highly civilised nation, do all in our power to prevent this elimination by building asylums and hospitals, and our medical men exert themselves to the very last to prolong life, even in fatal diseases. In order that a nation may grow stronger, means must be provided for the survival of the strongest only. A warlike nation is weakened as much by the feeble men who remain at home and propagate their kind as by the loss of the strongest in battle.

Interesting, though startling, is the fact that the increase of crime during the decade ending 1890, over that ending 1880, is from 1 to 299 per cent., and is to be seen in nearly every state and territory in the union. The number of prisoners in the United States in 1890 was 82,329, of which 901 were insane. The number of inmates of juvenile reformatories the same year was 14,846. The number of inmates of benevolent institutions, public and private, June 1, 1890, (not including hospitals for the insane, schools for the deaf and blind, or asylums for the feeble-minded,) was 112,263, of which 55,316 were males and 56,947 were females. The number of paupers was 73,045. The number of divorces in 1867 was 9,937 ; in 1886, 25,535, or a total of 328,716 in twenty years, which shows a greater percentage of increase than the increase of population. The expenditures for the care of the insane in the state of New York amount to $4,000,000 annually, a sum larger than for any other department.

The criminal expense of this country is enormous. Recent statistics show that there are 52 penitentiaries in this country and over 17,000 jails. The first cost of these buildings was $500,000,-000. The annual expense of these institutions is $100,000,000, and during the last year, for which statistics have been prepared, 900,000 people were incarcerated.

Erie county expended, during the last year, for the insane, $259,782.41; schools, $241,597.64.

These are a few statistics that go to show the prevalence and rapid increase of unfavorable social conditions. To simply give the names of inebriety, tuberculosis, syphilis, idiocy and epilepsy is sufficient to remind us of the fact that the burden we are carrying is almost overwhelming. Then, the first duty of a state or nation is, or should be, to protect the lives of coming generations, as well as the lives and property of those now living. It should also be its duty to teach its people to know how to obtain and preserve health, and compel the healthy ones to protect their offspring, by marrying the strong and vigorous only. In other words, "you who were fortunately born must be the keeper of your dangerous though perhaps unfortunate brother man, or he will overpower and ruin you." The *Express* of August 6, 1896, contained the following, which shows how voluminously (?) our editors will sometimes treat an ordinary (?) topic:

> The man who killed his wife and children in Kentucky this week came of a feeble-minded family. Yet a government which disdains to be paternal permitted him to marry and raise a family of his own.

The law protects us, or rather commands us to keep away from the contagion of scarlet fever and diphtheria, and compels us to immunise ourselves against the ravages of small-pox; and do we complain? Yes, there are a few who do, but they are the ones that always object to anything that is proposed for the general good. Education will not protect, for we find our best educated people are oftentimes the ones who display the most ignorance regarding their physical wellbeing. Ministers of the gospel, who are admittedly among our most learned men, generally exhibit more ignorance in regard to medicine and the physical condition of the women they marry than any other class. The introduction of the Jewish law of marriage and divorce reads as follows:

> Marriage is the most important and sacred of all domestic relations. It is the origin of all other relations of life, and forms the foundation of human society. Besides, it is a relation in which man's happiness for life is materially involved, and which serves to protect and promote moral purity.

A race remains characteristic and distinctive until immigration or a mixing with some other race causes it to change. Owing to the stringent canonical marriage laws of the Jews, backed by the almost unanimous sentiment of the people, it is seldom that a Jew is united in marriage to a Gentile. As a result, he remains a Jew no matter to what nation he may belong. He has endured many and lasting persecutions, but owing to the great care he exercises in regard to marriage, his progress has been constant and wonderful, and he now occupies the most conspicuous places, commercially, politically and intellectually.

The advancement of civilisation is a most difficult problem when there is such a constant struggle being made by the ignorant and vicious to lower it. Marriage certainly should not be thought of by parties who can bring their children nothing but abject poverty, for poverty is a great evil that tends to perpetuate itself and leads to the most reckless and demoralising marital relations. This may appear to be going a little too far, to deny the poor man the privilege of having a so-called home of his own, but I do not mean the poor man who has it in him to work and gradually improve his condition, but the one who is the natural mendicant, the one who would feel decidedly uncomfortable if he had a nickel ahead. This class of people, who revel in poverty and beget a numerous progeny, should be controlled as thoroughly as the diseased and the criminals. Imbeciles, confirmed epileptics and drunkards, those who have been insane more than once, habitual criminals and paupers should all be denied the right of procreation. In preventing those not qualified to marry, it should be remembered that if it prove a hardship to a few who would undoubtedly have a strong desire to do so, the greatest good to the greatest number must always be considered. The child takes his life from his parents; it is his heritage, his estate; he cannot pass it over to some one else; he can only lay it down when the angel of death comes to bear it away. The responsibility of such a condition is to be exceeded by one only, and that of the one who brought it into being. While the consent of the parties is universally deemed necessary to make the contract of marriage legal, this seems to be the only safeguard one has to protect him from serious consequences, the law cannot redress or remedy. Of course, there are plenty of laws for all the incidents arising from the relationship. There are also laws sufficient to protect your life and property, but none to protect you from a fate that may be worse than the loss of either or both. Just think of it! You who have sons and daughters blessed with a happy, healthy ancestry, do you ever think of the possible (I might say probable) fate that awaits them by marrying into a family whose ancestry is notoriously bad ? I am afraid not. This is usually the last consideration thought of, when it should be the first. Wealth and social position, no one will deny, are important factors in making up the sum total of domestic happiness, but how insignificant when compared with that great servitor of human happiness—*health.* Can a more discontented, unhappy and woeful picture of life be drawn than that of a young man, rich in the endowment of health and full of the life and ambition that attend such a condition, wedded to a woman who puts upon his children the stamp of ill-health and makes of his life a dismal failure? Such a union seems more terrible to me, and is fraught with more evil consequences, than that of any other contract which can be made.

Ingersoll says : "The true family is the result of the true marriage, and the institution of the family should, above all things, be preserved." Among the many good things to be found in Bellamy's Looking Backward is the following : "Over the unborn our power is that of God and our responsibility like His toward us. As we acquit ourselves toward them, so let Him deal with us." It has been said that "The lives of most men are in their own hands," but a greater truth is expressed when we say that the lives of future generations are in the hands of those now living. The physical as well as mental impress we make upon those who are to follow can not be over-estimated. If we are to be left perfectly free to follow out the impulse of passion, or the ambitious promptings to attain wealth or social position through the matrimonial gate, regardless of physical consequences, then I can see no relief for the great majority of our people, but perpetual ill-health and misery. Our schools, pulpits and periodicals might enlighten our boys and girls, but will they do it? And would it do any particular good if they did? I imagine the same physical misalliances would be formed as heretofore. Social misalliances are sometimes made, but the parties to them always know they are breaking the unwritten law of social ethics. It may seem hard to some not to be allowed to traverse the road the spirit of love and poetry direct ; but, when a social law is such a perfect barrier, why should not a carefully framed statute, calculated to bestow constantly increasing blessings, be made and executed?

Throughout Brazil there prevails a remarkable and self-imposed law in relation to matrimony. It is recognised among all the higher classes. The man who is about to marry is required to furnish a certificate from one or more physicians that he is free from disease of a certain character and that he is free also from all signs of any of the diseases which are liable to be transmitted to the offspring. Not only that, but the physicians consulted must testify that, as far as they can learn, there exists no reason to believe that the union will be other than in accord with the laws of sanitation. Such a self-imposed family law is certainly very creditable to our sister republic, and is a most worthy example for us to follow until we can have something better. Everywhere we see the hand of improvement, and many of the blessings and luxuries we enjoy today were not even in dreamland fifty, twenty-five or even five years ago. The march of civilisation was never so rapid as now, and the time must soon come when our enlightened nation will understand and fully appreciate the danger arising from the prudish manner in which we treat the marriage question, and will correct many of its abuses.

As an embodiment of an idea, which can be changed as experience may dictate, I would suggest that the state (or, better, the nation,) appoint a medical staff of three experts for every county in the state, to examine all boys and girls at the age of from twelve to fifteen, relative to their physical condition and family history, and a record be kept of all applicants. I would have three distinct classes. Class A—Those being physically and mentally sound, of good habits, and having no history of any hereditary disease (not mentioned in this paper as prohibitory to marriage), for at least three preceding generations. Class B— Same qualifications as in Class A, but family history to descend to grandparents only. Class C must necessarily be applied to all those not included in Classes A and B. Certificates for Classes A and B should be granted by the medical examiners. No one should be allowed to marry outside of the class to which he or she belongs, and those having certificates should be required, when about to marry, to make an affidavit that they are free from all communicable diseases and are temperate in their habits. This would tend to make Classes A and B constantly stronger and better, and a growing desire to be so. Class C, at first, would greatly predominate, but a few generations would suffice to make it the smallest. In those possessing hereditary taints, and deprived of the privilege of mating with the healthy, nature soon solves the problem by eliminating them. Heretofore the surplus population has been taken care of by wars and epidemics, but now, as the tendency of our advanced civilisation is to arbitration and sanitation, the population of the more enlightened nations must necessarily increase more rapidly than ever before. As society is at present constituted and controlled, the unhealthy and vicious class is increasing more rapidly than the more desirable one. In the days of Malthus the danger lay in the population increasing more rapidly than the means of subsistence, which meant a displacement of the weaker and more unfortunate members of society. Now this danger no longer threatens, but a greater one in the survival and overwhelming increase of imperfect physical and mental beings. By laws carefully made and judiciously administered, society can be gradually improved and danger averted.

37 ELMWOOD AVENUE.

